# Deciphering CAPTCHAs: What a Turing Test Reveals about Human Cognition

**DOI:** 10.1371/journal.pone.0032121

**Published:** 2012-03-01

**Authors:** Thomas Hannagan, Maria Ktori, Myriam Chanceaux, Jonathan Grainger

**Affiliations:** Laboratoire de Psychologie Cognitive, CNRS and Aix-Marseille University, Marseille, France; French National Centre for Scientific Research, France

## Abstract

Turning Turing's logic on its head, we used widespread letter-based Turing Tests found on the internet (CAPTCHAs) to shed light on human cognition. We examined the basis of the human ability to solve CAPTCHAs, where machines fail. We asked whether this is due to our use of slow-acting inferential processes that would not be available to machines, or whether fast-acting automatic orthographic processing in humans has superior robustness to shape variations. A masked priming lexical decision experiment revealed efficient processing of CAPTCHA words in conditions that rule out the use of slow inferential processing. This shows that the human superiority in solving CAPTCHAs builds on a high degree of invariance to location and continuous transforms, which is achieved during the very early stages of visual word recognition in skilled readers.

## Introduction

While browsing the Internet one is regularly annoyed by requests to demonstrate that one is not a robot. The most familiar of these CAPTCHAs - Completely Automated Public Turing test to tell Computers and Humans Apart [1] - ask of us to type in some sequence of distorted but common characters. They are designed so that humans can rise to the challenge quite accurately in a matter of seconds, while silicon-based algorithms will fail almost certainly unless prohibitively vast computational resources are summoned. Computer scientists might blush at how little is currently needed to draw the line - some character deformation and cluttering in an adversarial background has proved to be sufficient. But equally or more humbling is that we have very little idea how humans can actually solve CAPTCHAs. Here we examined the basis of this operationally defining human ability.

CAPTCHAs are telling us something about the way humans represent and process strings of letters - what we will call orthographic processing [2]. First they inform us about what the system is not: it is apparently not like the powerful and sophisticated algorithms that are kept at bay by these challenges, which however often use expensive feature extraction methods, supervised Markov models, or computationally greedy lexical searches through directed letter graphs [3]. Second, we are learning just what and how much distortion the system can take while still remaining in the comfort zone of fast and accurate responding.

Human superiority in solving CAPTCHAs could be due to at least two factors. One, favoured by our subjective experience, could involve slow inferential processes to make explicit guesses on letter identities in the face of ambiguous bottom-up information, perhaps not unlike letter-by-letter reading. Another possibility however is that our extensive reading experience, often in difficult conditions (e.g., handwritten text), could have helped us develop an automatic system for orthographic processing that is highly tolerant to noise and shape variations in the input. Here we eliminated the possible use of slow inferential processes by presenting CAPTCHAs as prime stimuli in a masked priming experiment. Prime stimuli are presented very briefly and immediately before a clearly visible target stimulus (a real word, e.g., TABLE, or a nonsense string of letters - a “nonword”, e.g., TOBLE) that participants must classify as being a word or not [4]. This paradigm has become the “gold standard” in investigations of the fast-acting automatic processes involved in skilled human reading [5]. In our experiment, target stimuli were presented in normal print, and prime stimuli (which could be the same word/nonword as the target, or a different word/nonword) were presented either as CAPTCHAs or in normal print. The relative size of priming effects obtained from CAPTCHA primes versus primes in normal print will indicate the extent to which our CAPTCHA stimuli were processed automatically.

## Results

2×2 repeated-measures ANOVAs with relatedness (Related, Unrelated) and prime type (Print, CAPTCHA) as factors were run separately on mean correct RTs and error rates, for word and nonword targets (i.e., a total of 4 independent ANOVAs). An inverse transformation was applied to the RT data to reduce the influence of skewness and outliers [6], and analyses were then carried out by participants (F1 statistic) and by items (F2 statistic). ANOVAs performed on RTs trimmed by using an arbitrary cut-off of 2.5 standard deviations for outliers yielded the same results. Results on mean inverse RTs to word targets revealed a main effect of relatedness (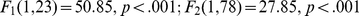), a main effect of prime type by participants (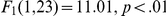) and a marginal effect of prime type by items (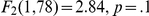). The interaction between relatedness and prime type was significant (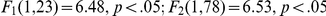), driven by an even more significant effect of relatedness for printed primes (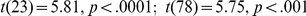) than for CAPTCHA primes (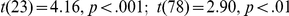). Analyses on mean error rates to word targets revealed a main effect of relatedness (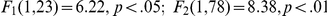), no effect of prime type (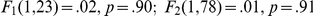) and no interaction between the two factors (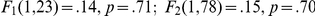). Overall, participants produced less errors to words following a related compared to an unrelated prime. No significant effects were revealed by the analyses performed on the data for nonword targets. The absence of priming effects for nonword targets is a standard result in masked priming lexical decision, as reviewed in [5], which also motivates independent ANOVAs for words and for nonwords.

## Discussion

**Figure 1:**
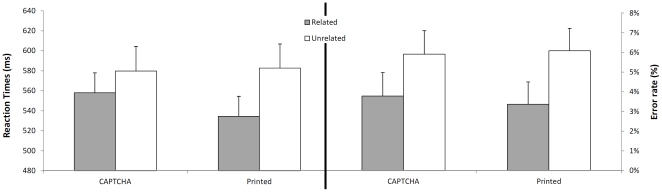
Evidence for automatic processing of CAPTCHA primes. Response times and percent errors for word targets as a function of prime condition: CAPTCHA prime vs. prime in normal print; prime is the same word as the target (grey) vs. prime is a different word (white). Error bars indicate the between-participants standard error of the mean for each condition. Statistical analyses were carried out on inverse response times.

Our results show that without rising to the level of normal printed words, word CAPTCHAs are remarkably efficient primes that generate a quite large and significant facilitation both in reaction times and in error rates (see Figure 1). Given the absence of visual overlap between primes and targets (primes and targets had different sizes, and different cases), participants must have extracted orthographic information from CAPTCHA stimuli under conditions that eliminated the use of slow inferential processes. This shows an ability of the human visual word recognition system that complements reports of strong facilitation with so-called “leet” primes (e.g. M4T3R14L-MATERIAL, [7]) or with handwritten primes [8]. Indeed our results strongly suggest that the human superiority in solving CAPTCHAs is at least partly due to what could be a more generic type of tolerance, not only to the alteration of selected letters or to handwritten character variations, but rather to global continuous input transforms and small letter rotations –in line with recent brain imaging results on rotated word recognition [9].

The flip-side of these findings is to hint at what might be valuable strategies for automatic text processing algorithms and cognitive models of reading alike, suggesting that a neuromorphic system should not emphasize slow computations (for instance direct searches in the lexicon) but rather might want to be primarily constrained by the very rapid resolution of subsets of letters under a variety of continuous transforms and rotations. This could possibly be achieved by combining both the letter-based and the bigram-pruning strategies proposed in [3]: first building fast “shape context representations” for individual letters, that indeed seem to possess the right invariant properties, and from then determining the most likely bigrams in the sequence, to ensure a drastic pruning of the lexical search space.

## Materials and Methods

### Participants

24 participants recruited from the undergraduate and postgraduate populations at Aix-Marseille University took part in the study. All were native French speakers and reported normal or corrected-to-normal vision.

### Design and Stimuli

A repeated-measures design was employed in which the three independent variables were Lexicality (words and nonwords), Prime Type (CAPTCHA and printed) and Relatedness (related and unrelated). Mean response time to correct responses and response accuracy in the lexical decision task were measured. Prime stimuli comprised 160 CAPTCHA and their equivalent 160 printed letter strings, half of which spelled familiar French words (5–10 letters long) and the other half readable nonwords (5–9 letters long). First, CAPTCHA stimuli were drawn from the reCAPTCHA website [10] ensuring that these contained only lowercase letters. All of the CAPTCHA stimuli showed a global continuous wavelike distortion in shape, and low quality letters tilted by at most 45 degrees from the vertical meridian. Printed primes were then matched to CAPTCHA primes as for identity and letter size. Target stimuli were the printed words and nonwords in uppercase letters but in a smaller font size than the prime stimuli so as to minimize visual overlap between the two. In the related condition the identity of the prime and target was the same. In the unrelated condition prime and target identities differed but were closely matched for letter string length. Stimuli were counterbalanced into four different lists of 160 trials with different pseudo-randomizations using the constraints that each target stimulus appeared once in each list and was paired with all the different prime conditions across the lists. In each list each experimental condition was equally represented (i.e., 20 repetitions). Following a practice session of eight trials, participants were assigned to one list of trials in a counterbalanced order. In each list, trials were presented randomly.

### Procedure

**Figure 2:**
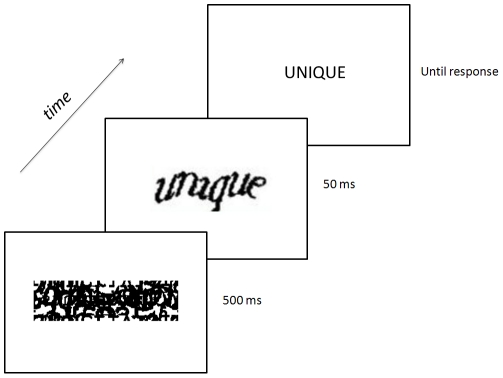
Masked priming lexical decision with CAPTCHA primes. On any trial, participants were exposed to a masking stimulus during 500 ms, followed by a prime for 50 ms in one of four conditions (repetition CAPTCHA prime condition depicted), and immediately followed by the target for a maximum of 1000 ms, which could be a word or a nonword (word trial depicted). Participants responded (“Word” or “Nonword”) using dedicated keys on the keyboard.

A masked priming lexical decision task was used. Participants were run individually in a sound-attenuated room. Each participant sat 82 cm in front of a 20″ monitor. The trial sequence of the experiment is illustrated in Figure 2. Each trial began with the presentation of a mask in the middle of the screen for 500 ms. Masks were designed by random scrambling, rotating and superimposing of CAPTCHA features. The mask was replaced at the same location with a prime for 50 ms that varied in type (CAPTCHA or lowercase print), and relatedness to the target (either the same or unrelated). The target stimulus then appeared in uppercase print and varied in lexicality (word or nonword). The target remained on the screen until participants' response. Participants were asked to indicate as quickly and as accurately as possible whether the target stimulus spelled a French word or not by pressing a response key in their right or left hand, respectively. The next trial followed a 1000 ms blank screen interval. E-Prime Version 2.0 controlled the randomization and presentation of the stimuli and logged the type of response and its latency. The experiment lasted approximately 15 minutes. Informed written consent was obtained from each participant before the experiment. This research (European Research Council #230313), including the method of consent, was approved by the internal review board of the Université de Provence.

## References

[1] von Ahn L, Blum M, Hopper N, Langford J (2003). Captcha: Using hard ai problems for security.. Advances in Cryptology, Eurocrypt.

[2] Grainger J (2008). Cracking the orthographic code: An introduction.. Language and Cognitive Processes.

[3] Mori G, Malik J (2003). Recognizing objects in adversarial clutter: Breaking a visual captcha..

[4] Forster KI, Davis C (1984). Repetition priming and frequency attenuation in lexical access.. Journal of Experimental Psychology: Learning, Memory, and Cognition.

[5] Forster KI (1998). The pros and cons of masked priming.. Journal of Psycholinguistic Research.

[6] Ratcliff R (1993). Methods for dealing with reaction time outliers.. Psychological Bulletin.

[7] Perea M, Duabeitia JA, Carreiras M (2008). R34d1ng w0rd5 w1th numb3r5.. Journal of Experimental Psychology: Human Perception and Performance.

[8] Gil-López C, Perea M, Moret-Tatay C, Carreiras M (2011). Can masked priming effects be obtained with handwritten words?. Atten Percept Psychophys.

[9] Cohen L, Dehaene S, Vinckier F, Jobert A, Montavont A (2008). Reading normal and degraded words: contribution of the dorsal and ventral visual pathways.. Neuroimage.

[10] von Ahn L, Maurer B, McMillen C, Abraham D, Blum M (2004). recaptcha: Human-based character recognition via web security measures.. Science September.

